# Get Rid of Nonlocality from Quantum Physics

**DOI:** 10.3390/e21080806

**Published:** 2019-08-18

**Authors:** Andrei Khrennikov

**Affiliations:** International Center for Mathematical Modeling in Physics and Cognitive Sciences, Linnaeus University, SE-351 95 Växjö, Sweden; Andrei.Khrennikov@lnu.se

**Keywords:** Bell type inequality, nonlocality, action at a distance, incompatibility, Landau identity, complementarity principle, Bell operator, squared Bell operator, Hertz-Boltzmann Bild methodology, prequantum classical statistical field theory

## Abstract

This paper is aimed to dissociate nonlocality from quantum theory. We demonstrate that the tests on violation of the Bell type inequalities are simply statistical tests of local incompatibility of observables. In fact, these are tests on violation of *the Bohr complementarity principle.* Thus, the attempts to couple experimental violations of the Bell type inequalities with “quantum nonlocality” is really misleading. These violations are explained in the quantum theory as exhibitions of incompatibility of observables for a single quantum system, e.g., the spin projections for a single electron or the polarization projections for a single photon. Of course, one can go beyond quantum theory with the hidden variables models (as was suggested by Bell) and then discuss their possible nonlocal features. However, conventional quantum theory is local.

## 1. Introduction

As is well known, the original EPR-argument [1] was fundamentally coupled with the Bohr complementarity principle [2,3,4] (see Section 6.1). Einstein, Podolsky, and Rosen reasoned against the completeness of quantum mechanics (QM) by showing that the “elements of reality” corresponding to two incompatible observables (e.g., position and momentum) can be assigned to the same physical system. However, their argument was purely theoretical (even merely philosophical) and it was impossible to check the EPR-statement experimentally. Bohr pointed to the latter in his reply to Einstein [5]; he considered the EPR-argument as metaphysical. Although nonlocality was mentioned in the EPR-paper, it was considered as just a possible alternative to incompleteness of QM.

Nonlocality was emphasized for the first time by by Bohm. It was elevated by Bell (who admired Bohmian mechanics) through the argument based on violation of Bell’s inequality [6,7,8].

Our aim is to perform the *genuine quantum mechanical analysis of the derivation of the CHSH-inequality* considered as an inequality for correlations of quantum observables—*the quantum CHSH-inequality*. Thus, we do not try to go beyond QM. We are interested in the quantum mechanical interpretation of experimental violation of the CHSH-inequality. We show that, in fact, the degree of violation is straightforwardly coupled to the degree of incompatibility of observables, the norms of commutators. In particular, in the scenario with two spatially separated systems, these are tests of local incompatibility, i.e., *incompatibility of observables on a single subsystem of a compound system* (Theorem 1, Section 4.2).

We remark that the CHSH-inequality [9] is derived for classical correlations expressed in the framework of hidden variables (established by Bell [6,7,8]) and by using the calculus of classical probabilities (the Kolmogorov probability theory). The quantum CHSH-inequality is derived for quantum correlations by using the operator formalism.

We stress that the CHSH combination of correlations,

(1) $$\langle B\rangle =\frac{1}{2}\left[\langle A_{1}B_{1}\rangle +\langle A_{1}B_{2}\rangle +\langle A_{2}B_{1}\rangle -\langle A_{2}B_{2}\rangle \right],$$

has three different interpretations:

- Classical (hidden variables) correlations, ${\langle B\rangle }_{\mathrm{CL}}.$
- Experimental correlations, ${\langle B\rangle }_{\mathrm{EXP}}.$
- Quantum mechanical correlations, ${\langle B\rangle }_{\mathrm{QM}}.$

Following Bell, one can compare the classical theoretical quantity ${\langle B\rangle }_{\mathrm{CL}}$ with its experimental counterpart ${\langle B\rangle }_{\mathrm{EXP}}.$ The majority of the quantum foundation and information community proceeds in this way. This way leads to operating with the notion of nonlocality and action at a distance.

*Is there any reason to couple this mysticism with quantum theory?*

It is more natural to start with the quantum theoretical analysis of quantity ${\langle B\rangle }_{\mathrm{QM}}.$ It is easy to explain under what circumstances it can be bounded by 1 (or exceed 1). (Thus so-called classical bound has the purely quantum origin.) This quantum mechanical explanation is purely local. Thus, it is really incorrect in quantum theory to speak about its nonlocality or associate with it any kind of action at a distance.

It is well known that (by the complementarity principle) it is impossible to measure jointly two spin coordinates for electron. Therefore, ${\langle B\rangle }_{\mathrm{QM}}$ can exceed 1. If somebody does not believe in this prediction of QM, it would be natural to check violation of the principle of complementarity for a single electron (or photon), e.g., to check violation of the Heisenberg uncertainty relation (in the form of the Robertson inequality).

As emphasized above, here I proceed by using solely the formalism of QM, cf. with probabilistic analysis of the incompatibility interpretation of the Bell type inequalities in [10,11,12,13,14,15,16,17,18,19,20,21,22,23,24] and especially [17]— the probabilistic version of the present paper. See also the recent preprint of Griffiths [25], where incompatibility of quantum observables is emphasized; see the recent works of Boughn [26,27], where the nonlocality viewpoint on quantum theory is critically analyzed and the role of the ontological vs. information interpretations of the wave function in discussions on “quantum nonlocality” is emphasized.

Foundational issues such as the complementarity principle, incompatibility, nonlocality, realism, and hidden variables, are discussed in more detail in Section 6.

## 2. Measuring the Degree of Incompatibility via the CHSH-Test

We show that the degree of violation the quantum CHSH-inequality can be considered as a measure of incompatibility in two pairs of quantum observables, $A_{1},A_{2}$ and $B_{1},B_{2}.$ This is the simple consequence of the *the Landau identity* [28,29] (see Equation (8)). In quantum theory incompatibility is mathematically expressed as noncommutativity. Thus, by testing incompatibility, we test the degree of noncommutativity, or in other words, the “magnitudes" of observables corresponding to commutators,

(2) $${\hat{M}}_{A}=i\left[{\hat{A}}_{1},{\hat{A}}_{2}\right],\;{\hat{M}}_{B}=i\left[{\hat{B}}_{1},{\hat{B}}_{2}\right].$$

We use the hat-symbol to denote operators.

The incompatibility-magnitude can be expressed via the maximal value of averages of commutator-operators, i.e., by their norms, for example,

(3) $$\underset{∥\psi ∥=1}{\sup}\left|\langle \psi \right|{\hat{M}}_{A}|\psi \rangle |=∥{\hat{M}}_{A}∥.$$

By interpreting quantity $\langle \psi |{\hat{M}}_{A}|\psi \rangle$ as the theoretical counterpart of experimental average ${\langle M_{A}\rangle }_{\psi }$ of observable $M_{A},$ we can measure experimentally the incompatibility-magnitude, i.e., norm $∥{\hat{M}}_{A}∥$ from measurements of commutator-observable $M_{A}.$ The main foundational problem is that measurement of such commutator-observables is challenging. Recently some progress was demonstrated on the basis of weak measurements, but generally we are not able to measure commutator-quantities.

We remark that (from the quantum mechanical viewpoint) the CHSH-test estimates the product of incompatibility-magnitudes for the *A*-observables and *B*-observables, i.e., the quantity $∥{\hat{M}}_{A}∥∥{\hat{M}}_{B}∥.$ However, by considering the *B*-observables as axillary and selecting them in a proper way (for example, such that the *B*-commutator is a simple operator), we can use the CHSH-test to get the experimental value for the incompatibility-magnitude $∥{\hat{M}}_{A}∥.$

## 3. Incompatibility as Necessary Condition of Violation of Quantum CHSH-Inequality

### 3.1. General Case: Without Referring to the Tensor Product Structure

Consider the Bohm–Bell type experiments. Four observables $A_{1},A_{2},B_{1},B_{2}$ taking values ±1 are considered. It is assumed that observables in each pair $A_{i},B_{j},i,j=1,2,$ can be measured jointly, i.e., *A*-observables are compatible with *B*-observables. However, the observables in pairs $A_{1},A_{2}$ and $B_{1},B_{2}$ are incompatible, i.e., they cannot be jointly measured. Thus, probability distributions $p_{A_{i}B_{j}}$ are well defined theoretically in QM and they can be verified experimentally; probability distributions $p_{A_{1}A_{2}}$ and $p_{B_{1}B_{2}}$ are not defined in QM and, hence, the question of their experimental verification does not arise.

We consider quantum observables represented by Hermitian operators. In QM, mathematical compatibility is represented by commutativity of operators, i.e., in the Bohm–Bell type experiments

(4) $$[{\hat{A}}_{i},{\hat{B}}_{j}]=0,i,j=1,2,$$

and generally $\left[{\hat{A}}_{1},{\hat{A}}_{2}\right]\neq 0,\;\left[{\hat{B}}_{1},{\hat{B}}_{2}\right]\neq 0.$ The quantum theoretical CHSH-correlation function has the form:

(5) $$\langle B\rangle =\frac{1}{2}\left[\langle {\hat{A}}_{1}{\hat{B}}_{1}\rangle +\langle {\hat{A}}_{1}{\hat{B}}_{2}\rangle +\langle {\hat{A}}_{2}{\hat{B}}_{1}\rangle -\langle {\hat{A}}_{2}{\hat{B}}_{2}\rangle \right].$$

(Here and everywhere below the index QM pointing to the quantum formalism is omitted.)

It is compared with the experimental CHSH-correlation function.

In the quantum framework, the CHSH-correlation function can be expressed with the aid of the Bell-operator:

(6) $$\hat{B}=\frac{1}{2}\left[{\hat{A}}_{1}\left({\hat{B}}_{1}+{\hat{B}}_{2}\right)+{\hat{A}}_{2}\left({\hat{B}}_{1}-{\hat{B}}_{2}\right)\right]$$

as

(7) $$\langle B\rangle =\langle \psi |\hat{B}|\psi \rangle .$$

By straightforward calculation, one can derive at the Landau identity:

(8) $${\hat{B}}^{2}=I-\left(1/4\right)\left[{\hat{A}}_{1},{\hat{A}}_{2}\right]\left[{\hat{B}}_{1},{\hat{B}}_{2}\right].$$

Thus, if *at least one of the commutators* equals to zero, i.e.,

(9) $$[{\hat{A}}_{1},{\hat{A}}_{2}]=0,$$

or

(10) $$[{\hat{B}}_{1},{\hat{B}}_{2}]=0,$$

then the following inequality holds:

(11) |〈B〉|≤1.

To derive this inequality, we used solely the quantum formalism. The inequality is the consequence of compatibility for at least one pair of observables, $A_{1},A_{2}$ or $B_{1},B_{2}.$ Thus, although formally Equation (11) coincides with the standard CHSH-inequality, it has totally different meaning. It is better to call Equation (11) *the quantum CHSH inequality.*

Thus, *compatibility of the A-observables or the B-observables is sufficient for validity of the quantum CHSH-inequality* (for all quantum states) or in other words *conjunction of incompatibilities of the A-observables and the B-observables is the necessary condition for its violation* (for some quantum state).

### 3.2. Compound Systems

States of a compound quantum system $S=(S_{A},S_{B})$ are represented in tensor product $H_{AB}=H_{A}⊗H_{B}$ of the state spaces $H_{A}$ and $H_{B}$ of subsystems $S_{A}$ and $S_{B}.$ Observables are given by operators

(12) $${\hat{A}}_{i}={\hat{A}}_{i}⊗I,\;{\hat{B}}_{i}=I⊗{\hat{B}}_{i},$$

where Hermitian operators ${\hat{A}}_{i}$ and ${\hat{B}}_{i}$ act in $H_{A}$ and $H_{B},$ respectively. They represent observables $A_{i},B_{i}$ on subsystems $S_{A},S_{B}$ of S. For spatially separated systems, we call them *local observables*.

This tensor representation automatically implies commutativity of operators ${\hat{A}}_{i}$ with operators ${\hat{B}}_{j},$ i.e., Equation (4) holds. We remark that the mathematical condition of incompatibility is reduced to condition $\left[{\hat{A}}_{1},{\hat{A}}_{2}\right]\neq 0\;\mathrm{and}\;\left[{\hat{B}}_{1},{\hat{B}}_{2}\right]\neq 0.$ For spatially separated systems, it is natural to call incompatibility of the observables on $S_{A}$ (on $S_{B}$) *local incompatibility*. Section 3.1 implies that *conjunction of local incompatibilities* is the necessary condition for violation of the quantum CHSH-inequality.

We remark that the mathematical formalism of this section is applicable to description of any kind of observables “respecting” the tensor product structure of observables. A physical system *S* need not be composed of two physical subsystems.

## 4. Incompatibility as Sufficient Condition of Violation of Quantum CHSH-Inequality

### 4.1. General Case: Without Referring to the Tensor Product Structure

Assume that *A*-observables as well as *B*-observables are incompatible, i.e., corresponding operators do not commute:

(13) $$\left[{\hat{A}}_{1},{\hat{A}}_{2}\right]\neq 0\;\mathrm{and}\;\left[{\hat{B}}_{1},{\hat{B}}_{2}\right]\neq 0,$$

i.e.,

(14) $${\hat{M}}_{A}\neq 0\;\mathrm{and}\;{\hat{M}}_{B}\neq 0,$$

where ${\hat{M}}_{A}=i\left[{\hat{A}}_{1},{\hat{A}}_{2}\right],\;{\hat{M}}_{B}=i\left[{\hat{B}}_{1},{\hat{B}}_{2}\right].$ It is important to note that $[{\hat{M}}_{A},{\hat{M}}_{B}]=0.$ We can write the Landau identity in the form

(15) $${\hat{B}}^{2}=I+\left(1/4\right){\hat{M}}_{AB},$$

where ${\hat{M}}_{AB}={\hat{M}}_{A}{\hat{M}}_{B}.$ If $M_{AB}=0,$ then, despite the incompatibility condition in Equation (13), the QCHSH-inequality cannot be violated. We proceed under condition

(16) $${\hat{M}}_{AB}\neq 0.$$

In our framework, this condition is not so restrictive. We consider the quantum CHSH-inequality as a statistical test of incompatibility. It is natural to estimate the degree of incompatibility in one pair of observables, e.g., in the *A*-pair. In this approach, the *B*-pair plays the axillary role and we can freely play with its selection. To obtain the condition in Equation (16), it is sufficient to select *B*-operators in such a way that the operator ${\hat{M}}_{B}$ is invertable. We especially highlight the case of compound systems (see Section 3.2). Here incompatibility of the *A*-observables and the *B*-observables, see Equation (14), automatically implies the condition in Equation (16)

Under the condition in Equation (16), there exists some common eigenvector $\psi_{AB}$ such that $M_{A}\psi_{AB}=\mu_{A}\psi_{AB},M_{B}\psi_{AB}=\mu_{B}\psi_{AB}$ and both eigenvalues are nonzero.

Suppose that $\mu_{A}>0$ and $\mu_{B}>0.$ Then, this $\psi_{AB}$ is an eigenvector of operator ${\hat{B}}^{2}$ with eigenvalue $\left(1+\mu \right)>1,\mu =\mu_{A}\mu_{B}.$ Hence, $∥{\hat{B}}^{2}∥\geq \left(1+\mu \right)>1$ and

$$1<\left(1+\mu \right)\leq ∥{\hat{B}}^{2}∥=∥\hat{B}{∥}^{2}.$$

Since $\hat{B}$ is Hermitian, we have

$$∥\hat{B}∥=\underset{∥\psi ∥=1}{\sup}\left|\langle \psi \right|\hat{B}|\psi \rangle |.$$

Finally, we get that

$$\underset{∥\psi ∥=1}{\sup}\left|\langle \psi \right|\hat{B}|\psi \rangle |>\sqrt{1+\mu }>1.$$

Thus, there exist pure quantum states such that the QCHSH-inequality is violated.

Now, suppose that $\mu_{A}>0,$ but $\mu_{B}<0.$ To change the sign of $\mu_{B},$ it is sufficient to interchange the *B*-observables.

Thus, *conjunction of incompatibilities of the A-observables and the B-observables constrained by Equation (16) is sufficient for violation of the quantum CHSH-inequality.*

### 4.2. Compound Systems

Here, $H=H_{A}⊗H_{B}$ and ${\hat{A}}_{j}={\hat{A}}_{j}⊗I,{\hat{B}}_{j}=I⊗{\hat{B}}_{j},$ where Hermitian operators ${\hat{A}}_{j}$ and ${\hat{B}}_{j}$ act in $H_{A}$ and $H_{B},$ respectively.

#### 4.2.1. Incompatibility as Necessary and Sufficient Condition of Violation of the Quantum CHSH-Inequality

Here, the joint incompatibility-condition in Equation (13) is equivalent to incompatibility of observables on subsystems:

(17) $${\hat{M}}_{A}=i\left[{\hat{A}}_{1},{\hat{A}}_{2}\right]\neq 0\;\mathrm{and}\;{\hat{M}}_{B}=i\left[{\hat{B}}_{1},{\hat{B}}_{2}\right]\neq 0.$$

We have ${\hat{M}}_{AB}={\hat{M}}_{A}{\hat{M}}_{B}={\hat{M}}_{A}⊗{\hat{M}}_{B}.$ As mentioned above, constraint ${\hat{M}}_{AB}\neq 0$ is equivalent to Equation (17). Section 3.1 implies that *conjunction of local incompatibilities* is the sufficient condition for violation of the quantum CHSH-inequality. Thus, we obtain:

**Theorem** **1** (Local incompatibility criteria of QCHSH-violation)**.** *Conjunction of local incompatibilities is the necessary and sufficient condition for violation of the quantum CHSH-inequality.*

#### 4.2.2. Eigenvectors of the Bell Operator and Its Square

Consider the eigenvector consideration of Section 4.1. The vector $\psi_{AB}=\psi_{A}⊗\psi_{B},$ where $\psi_{A}\in H_{A},\psi_{B}\in H_{B},$ and ${\hat{M}}_{A}\psi_{A}=\mu_{A}\psi_{A},{\hat{M}}_{B}\psi_{B}=\mu_{B}\psi_{B}.$ We assume that $\mu =\mu_{A}\mu_{B}>0.$ Then,

$$\langle \psi_{A}⊗\psi_{B}|{\hat{B}}^{2}|\psi_{A}⊗\psi_{B}\rangle >1.$$

Thus, for the squared CHSH-observable ${\hat{B}}^{2},$ the one-boundary can be violated for separable states. Here, entanglement of *A* and *B* observables plays no role.

To be more illustrative, let us restrict consideration to finite dimensional Hilbert spaces. There can be found states Ψ and Φ such that

$$\max_{∥\psi ∥=1}\left|\langle \psi \right|{\hat{B}}^{2}\left|\psi \rangle \right|=\langle \Psi |{\hat{B}}^{2}|\Psi \rangle ,$$

$$\max_{∥\psi ∥=1}\left|\langle \psi \right|\hat{B}|\psi \rangle =\left|\langle \Phi \right|\hat{B}|\Phi \rangle .$$

The tricky thing is that generally Ψ≠Φ. The equality for norms, $∥\hat{B}∥=\sqrt{∥{\hat{B}}^{2}∥},$ does not imply equality of the max-optimization states.

Of course, max-states for B and $B^{2}$ are connected: the former can be represented as linear combinations of the latter (the feature of all operators with degenerate spectrum. (as shown in [30], max-states for B can be represented even as mixtures of max-states for $B^{2}$.)

In the experiments to violate the quantum CHSH-inequality, tremendous efforts were put to prepare ensembles of entangled states. The main reason for this is that the direct measurement of the observable represented by operator ${\hat{B}}^{2}$ is challenging. In Appendix B, we present the abstract analog of the Bell experiments treated as experiments to measure the degree of incompatibility. The tensor product structure is excluded and, in particular, an analog of entangled states related to measurement of an observable and its square is considered.

## 5. CHSH-Correlation Function as Measure of Incompatibility

We start with consideration of observables respecting the tensor product structure on the state space $H=H_{A}⊗H_{B}.$ Consider the eigenbases $\left(e_{Ak}\right)$ and $\left(e_{Bk}\right)$ of operators ${\hat{M}}_{A}$ and ${\hat{M}}_{B}$ (acting in $H_{A}$ and $H_{B},$ respectively) and the corresponding eigenvalues $\mu_{Aj},\mu_{Bj}.$

Let $∥{\hat{M}}_{A}∥=\max_{j}|\mu_{Aj}\left|=\right|\mu_{Ai_{a}}|,∥{\hat{M}}_{B}∥=\max_{j}|\mu_{Bj}\left|=\right|\mu_{Bi_{b}}|$ and let $\mu_{Ai_{a}}\mu_{Bi_{b}}>0.$ Then, $∥{\hat{B}}^{2}∥=\left(1+\mu_{Ai_{a}}\mu_{Bi_{b}}\right).$ Thus,

(18) $$b=∥\hat{B}∥=\sqrt{1+\frac{1}{4}∥\left[{\hat{A}}_{1},{\hat{A}}_{2}\right]∥\;∥\left[{\hat{B}}_{1},{\hat{B}}_{2}\right]∥},$$

where ${\langle B\rangle }_{\psi }$ is given by Equation (5); *b* is the maximal possible value of CHSH-correlations. If eigenvalues $\mu_{Ai_{a}}$ and $\mu_{Bi_{b}}$ have different signs, then we interchange the *B*-observables.

From Equation (18), we get that

$$∥\left[{\hat{A}}_{1},{\hat{A}}_{2}\right]∥∥\left[{\hat{B}}_{1},{\hat{B}}_{2}\right]∥=4\left(b^{2}-1\right).$$

The norm of commutator can be considered as a measure of incompatibility. Thus, the CHSH-correlation function gives the possibility to check experimentally the product of degrees of incompatibility for the *A* and *B* observables.

One may consider this way of measuring of incompatibility as too tricky. However, typically, to measure commutator-observable and then its average is challenging (by “measuring commutator-observable”, we mean measuring observable represented mathematically by commutator operator scaled by *i*). Therefore, even such a tricky approach to this problem as measurement of the CHSH-correlation function deserves attention.

Now, consider *B*-observables as axillary. In this way, we are able to determine the degree of incompatibility for the *A*-observables by using some pair of axillary observables $B_{1},B_{2}.$ We can select the latter in such a a way that their commutator is a “good observable”, so that it can be easily measured for any state, thus its average and hence the norm can be determined. Then, we can measure incompatibility of observables $A_{1}$ and $A_{2}$ by using the formula:

(19) $$∥\left[{\hat{A}}_{1},{\hat{A}}_{2}\right]∥=4\left(b^{2}-1\right)/∥\left[{\hat{B}}_{1},{\hat{B}}_{2}\right]∥.$$

Why is the use of tensor product states so useful for measuring the degree of incompatibility? By spitting a system into two subsystems it is easy to check compatibility of *A* and *B* observables, thus the possibility to define the CHSH-correlation function which can be measured in experiment.

## 6. Foundational Questions

### 6.1. Bohr’s Complementarity Principle

Often, it is claimed that Bohr’s writings and, in particular, about the complementarity principle are very difficult for understanding. For example, in Schilpp’s volume [31], p. 674 (see also Plotnitsky [32], p. 108), one can find the following statement: “*Thus, Einstein was confessed, after decades of his exchanges with Bohr, that he was ‘unable to attain ... the sharp formulation ... [of] Bohr’s principle of complementarity”’.* This principle has the complex structure and composed of a few components. One of the problems is that typically this principle is reduced to just one of its components, namely, the incompatibility-component. Incompatibility has the most striking consequences for quantum theory and experiment. However, as separated from the body of the complementarity principle, incompatibility is difficult for understanding.

As emphasized in [17], the complementarity principle is in fact the principle of contextuality of quantities used in the quantum formalism, in the sense of coupling them to corresponding experimental contexts. Bohr did not use the notion “experimental context". He considered experimental conditions [2]:

*“Strictly speaking, the mathematical formalism of quantum mechanics and electrodynamics merely offers rules of calculation for the deduction of expectations pertaining to observations obtained under well-defined experimental conditions specified by classical physical concepts.”*

By using the notion of experimental context as the synonymous of Bohr’s experimental conditions we can present the complementarity principle as composed of the following components [17] (we remark that one has to be very careful by operating with the notion of contextuality. Nowadays, this notion is widely used in foundational discussion on the Bell type inequalities. In such discussions, the meaning of the notion contextuality does not coincide with Bohr’s contextuality, as taking into account the experimental con- text to explain the mechanism of generating the values of a quantum observable. From Bohr’s viewpoint, any single quantum observable is contextual. One may say that consideration of Bohr’s contextuality in parallel with Bell’s contextuality can be misleading. However, we can consider Bell’s contextuality simply as a very special case of Bohr’s contextuality).

- (B1): There exists the fundamental quantum of action given by the Planck constant *h*:
- (B2): The presence of *h* prevents approaching internal features of quantum systems.
- (B3): Therefore, it is meaningless (from the viewpoint of physics) to build scientific theories about such features.
- (B4): An output of any observable is composed of contributions from a system under measurement and the measurement device.
- (B5): Therefore, the complete experimental arrangement (context) has to be taken into account.
- (B6): There is no reason to expect that all experimental contexts can be combined. Therefore, there is no reason to expect that all observables can be measured jointly. Hence, there exist incompatible observables.

(B6) can be called the incompatibility principle; this is a consequence of (B4) and (B5). Typically, the complementarity principle is identified with (B6). However, such a viewpoint does not match Bohr’s understanding of the complementarity principle, as the combination (B1)–(B6).

This is the good place to remark that (B6) is very natural. The existence of incompatible experimental contexts is not surprising. Compatibility of all experimental contexts would be really surprising.

### 6.2. “Quantum Nonlocality”

We briefly discuss the notion of (non)locality.

#### 6.2.1. Relativistic Invariance

Everywhere in physics, besides the Bell inequality debates [6,7,8,9,30,33,34,35,36], locality is identified with the relativistic invariance of theory. Therefore, the statements on nonlocality of quantum theory can make the impression (and they do!) that there is something wrong with relativistic invariance. However, there is nothing wrong with relativistic invariance. Of course, QM (in particular, the Schrödinger equation) is not relativistically invariant and attempts to construct relativistically invariant QM (based on the Dirac equation) were not successful. However, *QM is an approximation of quantum field theory which is relativistically invariant* (see Bogolubov and Shirkov [37] and Haag [38] (especially Chapter 3, “Algebras of Local Observables and Fields”)). To complete the picture, we remark that there is a non-relativistic quantum field theory (see for example, book [39]).

#### 6.2.2. Hidden Variables and Action at a Distance

One can say that nonlocality is a consequence of “action at the distance” [6,7,8] (see, e.g., Shimony [40,41] and Jaeger [42,43] for the detailed presentation). This interpretation is based on the invention of hidden variables. However, the analysis presented in this paper shows clearly that, to proceed in this framework, *one has to start with rejection of the basic principle of QM, the complementarity principle.* It is not clear why violations of this principle should be sought for compound systems. Thus, before inventing hidden variables, it would be natural to find violations of say the Heisenberg uncertainty principle (in the form of Robertson inequality).

Moreover, the modern attempt to go beyond QM with hidden variables is too straightforward. Already in the 19th century, in the process of transition from Newtonian mechanics to classical field theory, physicists were confronted with the same problem as in the process of transition from classical physics to QM. It was resolved in the framework of Bild (image) methodology developed by Hertz and Boltzmann [44,45,46] (see Section 6.4 and papers [17,47,48]).

#### 6.2.3. Nonlocality = Violation of the Bell Type Inequalities.

The common comment to my talks is that per definition “quantum nonlocality” is a violation of the Bell type inequalities. However, this viewpoint is really misleading. If one recognizes that such violation is just a signature of incompatibility, then it is strange to speak about nonlocality, instead of complementarity.

### 6.3. Obscuring Incompatibility by Tensor Product Structure of Observables

As pointed out, we concentrate our analysis on the CHSH-inequality [9]. In contrast to the previous studies (see, e.g., [30,33,34,35,36]), we do not emphasize the role of the tensor product structure for the state space and observables. We proceed in the general framework and the tensor product model is just a special case of this framework. The common emphasis of the tensor product structure obscures the crucial role played by incompatibility of observables. Mathematically the crucial role of incompatibility-noncommutativity for violation of the CHSH-inequality was clarified already by Landau [28,29] (see also [30,33,34,35,36]). However, the mathematical calculations presented in these works did not lead to reinterpretation of violation of the CHSH-inequality.

I would like to emphasize the crucial role played by works of Landau [28,29]. In fact, Landau’s articles carried the same message as the present paper: the CHSH inequality is an experimental test of the principle of complementarity. (He even used the terminology “complementary observables”, instead of “incompatible observables”.) Unfortunately, his excellent mathematical work was not completed by an extended interpretational discussion. Surprisingly, nowadays, his works are practically forgotten (of course, qualified people are aware about papers [28,29]. However, generally, the members of the quantum foundational community practically never refer to these papers. During 20 years of debates on the Bell inequality in Växjö, I have never heard about them. I got to know about Landau’s works from E. Dzhafarov, an expert in mathematical psychology. It happened say eight years ago and I also ignored the complementarity message of Landau. I was content to enjoy his mathematics). I see two reasons for this:

1. Landau used the abstract framework of $C^{★}$-algebras and, for many “real physicists”, this was not so attractive.
2. He emphasized the novel way to derive the Tsirelson bound and typically this paper is considered as devoted to this derivation, i.e., its crucial component, coupling of Bell’s argument to Bohr’s principle of complementarity was completely ignored.

In the present paper, I select the intermediate strategy for representation. On the one hand, I do not just follow Landau using the language of $C^{★}$-algebras. On the other hand, I also do not want to follow the common path based on the tensor product representation. I proceed in the complex Hilbert space formalism, but generally without referring to the tensor product structure of operators. Mathematics is really simple. It is based on the interrelation of spetcral properties of the Bell operator B and its square $B^{2}$ (In fact, I have the impression that the essence of CHSH-test is this spectral interplay between the spectral properties of a Hermitian operator and its square. I try to present this vision in the abstract form in Appendix B).

### 6.4. Herz–Boltzmann Bild-Methodology of Science

It is surprising that not only Bell, but even Einstein, Bohr, and Heisenberg did not know about the works of Hertz and Boltzmann [44,45,46] on so-called “Bild” (image) methodology for physical theories. According to Hertz and Boltzmann, when speaking about a scientific theory, one has to specify its type: descriptive theory or observational theory. The crucial point is that a descriptive theory need not be straightforwardly coupled with theory of observations. By extending the Hertz–Boltzmann methodology to the quantum domain, we recognize that QM is an observational theory. Theories with hidden variables are of the descriptive type. The same observational theory can be based on a variety of descriptive theories. Bell’s type descriptive theories have very rigid coupling to QM, the observational theory. One can construct a variety of corresponding descriptive theories which are not constrained by the Bell type inequalities.

In this paper, we do not to discuss the Bild-methodology of Herz and Boltzmann [44,45,46] in much detail (see my recent article [48]). We only make the remark on the notion of realism. From the Bild-viewpoint, realism in physics as well as any other area of science is reduced solely to experimental facts. In QM, this is exactly Bohr’s point of view. Thus, the only realistic component of QM are outcomes of measurements (Bohr’s phenomena). Any physical theory (descriptive as well as observational) is only about human images of natural phenomena. At the same time, these images are created on the basis of human’s interaction with nature.

Neither Einstein nor Bohr was not aware of the works of Hertz and Bolzmann. (In any event, they never cited these works.) Both Einstein and Bohr identified descriptive and observational theories. In fact, the EPR-paper [1] can be considered as the message that QM is not a descriptive theory. However, at the same time, Einstein–Podolsky–Rosen dreamed of a descriptive theory with the straightforward coupling to observations. According to Hertz and Boltzmann, the latter is generally impossible. In his reply [5], Bohr tried to explain that QM is an observational theory and such things as the EPR elements of reality do not belong to its domain. However, nobody was aware about Hertz–Boltzmann distinguishing of descriptive and observational theories. Therefore their discussion can be compared to conversation of the blind with the deaf.

Finally, we refer to an example of descriptive theory coupled to QM (treated as an observational theory). This is *prequantum classical statistical field theory* (PCSFT), which was developed by the author of this paper and coauthors [49] (see Appendix C).

### 6.5. On Incompatibility Interpretation of the Bell Type Inequalities

In this paper, we analyze the CHSH-inequality and conclude that this is a test of the complementarity principle. It seems that this analysis can be extended to other Bell type inequalities. The crucial mathematical step in this analysis is derivation of the analogs of the Landau identity (see Hardy [33] for generalization of the CHSH inequality to an *N*-measurement scheme and Cereceda’s paper [34], where the very general case (including Mermin’s inequalities) was studied in very detail).

In Appendix A, we show (independently of results based on the Landau identity that incompatibility for at least one pair of observables is the necessary condition for violation of any type of the Bell type inequalities.

## 7. Conclusions

The point of Bell’s theorem is that a local hidden variables theory cannot reproduce the results of quantum theory. The implication is that only a nonlocal hidden variable theory (like Bohmian mechanics) can reproduce the correlations found in quantum theory (and in the real world). Here, clearly, “nonlocality” refers to hidden variables theories, not to quantum theory. The very common misconception is to (incorrectly) associate the term with quantum theory. (In particular, from this viewpoint, the comments of Aspect [50] and Wiseman [51] on the crucial experiments [52,53,54] are really misleading; cf. with the comment of Kupczynski [24] and the author of this paper [55]).) We hope that the argument presented in this paper has convincingly demonstrated that this association is wrong. The deeper message of this paper is that the Bell inequality can be reinterpreted as *a condition on the quantum compatibility of local observables.* If local commutators vanish, the correlations are bounded just as they are when hidden variables are assumed to be local. The Bell type inequalities have one interpretation for hidden variables theories (the classical case), and another lesser-known and very interesting one for quantum theory.

Consequently the outputs of experiments testing violation of the Bell type inequalities also can be interpreted in two different ways. The conventional interpretation is that these were classical vs. quantum physics tests. My interpretation is that such experiments were, in fact, the tests of local incompatibility of quantum observables. I claim that the latter interpretation does not diminish the foundational value of these breakthrough experiments [52,53,54,56,57]. Complementarity is the basic feature of quantum observables. Tests on this feature are of the great foundational importance. At the same time the conventional interpretation of these tests, local realism vs. quantum theory is, in fact, not so exciting. What is the meaning to test nowadays quantum against classical? The validity of quantum theory was confirmed by the huge body of experiments and technological applications.

We also point out that the Bell type experiments played the crucial role in development of quantum technology: creation of efficient sources of entangled systems and photo-detectors as well as transmission of quantum systems to long distances with minimal disturbance.

It is clear that to get rid of nonlocality from quantum theory is not a simple task. The present note is just a step towards the common acceptance of the local interpretation of QM.

This paper should not be considered as directed against attempts to go beyond QM, by introducing “hidden variables”. However, in such attempts, one has to take into account the basic principles of QM an especially the complementarity principle (see the recent article of Khrennikov and Alodjants [18]). One also has to take into account the lessons of 19th century physics in the period of transition from Newtonian mechanics to field theory (Section 6.4).

## Appendix A. Incompatibility as Necessary Condition for Violation of Any Bell Type Inequality

Consider a family of quantum observables $D_{1},\ldots ,D_{n}$ represented by Hermitian operators ${\hat{D}}_{1},\ldots ,{\hat{D}}_{n}.$ We restrict considerations to observables with discrete values; thus, operators have the purely discrete spectra. Denote by ${\hat{E}}_{i}\left(x\right)$ the orthogonal projector corresponding to the eigenvalue *x* of ${\hat{D}}_{i}.$

Suppose that the observables are pairwise compatible, i.e., $\left(D_{i},D_{j}\right)$ can be measured jointly for any quantum state ρ and jpd is well defined

(A1) $$p_{D_{i}D_{j}}\left(x,y;\rho \right)\equiv P\left(D_{i}=x,D_{j}=y;\rho \right).$$

In QM, compatibility is mathematically represented via commutativity of operators, i.e., $[{\hat{D}}_{i},{\hat{D}}_{j}]=0,$ and, hence, $[{\hat{E}}_{i}\left(x\right),{\hat{E}}_{j}\left(y\right)]=0.$ The quantum formalism gives the following formula for jpd (von Neumann [58]):

(A2) $$p_{D_{i}D_{j}}\left(x,y;\rho \right)=\mathrm{Tr}\;æE_{i}\left(x\right)E_{j}\left(y\right)=\mathrm{Tr}\;æE_{j}\left(y\right)E_{i}\left(x\right).$$

Now, we point to the really surprising feature of quantum measurement theory. If observables are pairwise compatible, i.e., each pair can be jointly measured with corresponding jpds $p_{ij}\left(x,y;\rho \right)$ given by Equation (A2), then they are also triple-wise compatible, quadruple-wise compatible and so on... Any family of observables, $D_{i_{1}},\ldots ,D_{i_{m}}$ can be jointly measured and the joint probability distribution is given by the formula:

(A3) $$p_{D_{i_{1}}\ldots D_{i_{m}}}\left(x_{1},\ldots ,x_{m};\rho \right)=\mathrm{Tr}\;æE_{i_{1}}\left(x_{1}\right)\ldots E_{i_{m}}\left(x_{m}\right).$$

On the left-hand side of this formula, one can take any permutation of indexes. This implies:

2⇒m: *pairwise compatibility ⇒ multiple compatibility.*

This is really astonishing. It is surprising that its specialty (from the general viewpoint of measurement theory) was not discussed in foundational literature.

We turn to Bell’s inequalities. Now, we are endowed with 2⇒m property of quantum observables.

Consider the most general Bell-type framework. There are *K* groups of quantum observables:

$$D^{k}=\left(D_{1}^{k},\ldots ,D_{N_{k}}^{k}\right),k=1,\ldots ,K.$$

Mathematically, they are represented by Hermitian operators:

$${\hat{D}}^{k}=\left({\hat{D}}_{1}^{k},\ldots ,{\hat{D}}_{N_{k}}^{k}\right).$$

Suppose that, for different k, observables are compatible, i.e., in the mathematical framework:

$$[{\hat{D}}_{i}^{n},{\hat{D}}_{j}^{m}]=0,n\neq m.$$

Thus, jpds $p_{i_{1}\ldots i_{K}}\equiv p_{D_{i_{1}}^{1}\ldots D_{i_{K}}^{K}}$ are well defined and, hence, covariations as well

$$\langle D_{i_{1}}^{1}\cdots D_{i_{K}}^{K}\rangle =\mathrm{Tr}\;æ{\hat{D}}_{i_{1}}^{1}\cdots {\hat{D}}_{i_{K}}^{K}=\sum x_{1}\cdots x_{K}\;p_{i_{1}\ldots i_{K}}\left(x_{1},\ldots ,x_{K}\right).$$

Consider some Bell-type inequality (e.g., the CHSH inequality or the Mermin inequality),

(A4) $$\sum_{i_{1}\ldots i_{K}}\;t_{i_{1}\ldots i_{K}}\;\langle D_{i_{1}}^{1}\ldots D_{i_{K}}^{K}\rangle +\mathrm{correlations}\;\mathrm{of}\;\mathrm{lower}\;\mathrm{orders}\leq c,$$

where $t_{i_{1}\ldots i_{K}}$ are some real constants. This inequality may be violated only if at least one pair of observables, say $(D_{i}^{n},D_{j}^{n}),$ is incompatible, i.e., in the mathematical formalism

(A5) $$[{\hat{D}}_{i}^{n},{\hat{D}}_{j}^{n}]\neq 0.$$

Otherwise, jpd exists and the inequality in Equation (A4) cannot be violated.

**Theorem** **A1.**
*Incompatibility is a necessary condition for violation of any Bell-type inequality.*

In the standard nonlocality discussions, it is assumed that there are *K* systems $S^{k},k=1,2,\ldots ,K,$ and observables $D^{k}$ are local observables for $S^{k}$. Endowed with this scheme, we analyze the possibility to violate the Bell-type inequality in Equation (A4). The necessary condition is that Equation (A5) holds true. This condition is local.

## Appendix B. “Entanglement” in the Absence of the Tensor Product Structure

In Section 4.2.2, we consider compound systems and discuss the well known fact that eigenvectors of operator ${\hat{B}}^{2}$ giving the max-value of its quadratic form can be selected as separable (non-entangled) states; they need not be eigenvectors of the Bell operator; its eigenvectors are linear combinations of the aforementioned separable states.

We want to show that the tensor product structure of states and operators is not crucial for the above consideration.

Consider any Hermitian operator $\hat{C}$ and its square ${\hat{C}}^{2}.$ Let *u* be an eigenvector of the latter, i.e., ${\hat{C}}^{2}u=\lambda u,\lambda >0,$ and let *u* is not an eigenvector of the former. Set $v=\hat{C}u/\sqrt{\lambda },$ i.e.,

(A6) $$\hat{C}u=\sqrt{\lambda }v,\;\hat{C}v=\sqrt{\lambda }u.$$

Set

(A7) $$\psi_{\pm }=u\pm v.$$

Then, $\hat{C}\psi_{\pm }=\pm \sqrt{\lambda }\psi_{\pm }.$ Thus, $\psi_{\pm }$ are eigenvectors of $\hat{C}.$

If the quadratic form of ${\hat{C}}^{2}$ approaches its max-value on eigenstate u/∥u∥, then the quadratic form of $\hat{C}$ approaches its max-value on eigenstate $\phi_{\pm }=\psi_{\pm }/∥\psi_{\pm }∥.$

States $\psi_{\pm }$ can be considered as *generalization of entangled states*, which is to say entangled with respect to operator $\hat{C}.$

This consideration can be coupled to measurement theory. Consider some quantum observable *D* represented by Hermitian operator $\hat{D}.$ (For simplicity, suppose that $\hat{D}\geq 0.$) Suppose that this observable is simple theoretically, by complex experimentally (Spectrum and eigenvectors of operator $\hat{D}$ can be easily found, but measurement of observable *D* is really challenging).

Consider also the observable *C* represented by Hermitian operator $\hat{C}\equiv \sqrt{\hat{D}}.$ Suppose that this observable is complex theoretically, but rather simple experimentally (The structure of spectrum and eigenvectors of operator $\hat{C}$ is complicated, but measurement design for *C* is straightforward).

We are interested in the following problem: *Find experimentally the upper bound for the average ${\langle D\rangle }_{\psi }$ of observable D with respect to all possible states.* We stress that we are interested in the experimental verification of a theoretical prediction of QM.

We can easily find state *u* corresponding to max-eigenvalue λ of operator $\hat{D}.$ Then, one of the max-states of operator $\hat{C}$ can be found with the aid of “*C*-entanglement”:

(A8) $$\phi_{+}=\left(u+\lambda^{-1/2}\hat{C}u\right)/∥u+\lambda^{-1/2}Cu∥.$$

Finally, we prepare an ensemble of systems in quantum state $\phi_{+}$ and perform *C*-measurement for these systems.

In the Bell-type scenario (for observables respecting the tensor product structure), $\hat{C}=\hat{B}$ is the Bell operator, $\hat{D}={\hat{B}}^{2}.$ In fact, the degree of incompatibility is encoded in the observable corresponding to operator $\hat{D}.$ However, its straightforward measurement would involve measurement of observables corresponding to commutators. The latter is challenging. At the same time, eigenstates of *D* have the simple tensor product structure (separable states). They can easily be found. Then, eigenstates of the Bell operator can be generated as superpositions of Equation (A8).

## Appendix C. Prequantum Classical Statistical Field Theory

The basic variables of PCSFT [49] are classical random fields defined on physical space. A random field can be considered as a function of two variables ϕ=ϕ(x;ω): *x* is the spatial variable (with three real coordinates); ω is a random parameter. We remark that random fields can be considered as random vectors valued in the complex Hilbert space $H=L_{2}\left(R^{3}\right)$ of square integrable complex valued functions.

The key point of this theory is that covariance operator *B* of random field ϕ is identified (up to normalization by trace) with the density operator of QM:

(A9) B→ρ=B/TrB.

The covariance operator is an element of the descriptive theory (PCSFT) and the density operator is the element of the observational theory (QM) (For a complex valued random field, its covariance operator *B* is a Hermitian positive operator with the finite trace. Thus, *B* has all features of a density operator, besides normalization Træ=1).

We remark that here the trace of field’s covariance operators equals to average of field’s energy:

(A10) $$\mathrm{Tr}B=E∥{CE\left(!\right)∥}^{2},$$

where *E* is mathematical expectation and

$${∥\phi \left(\omega \right)∥}^{2}=\int_{R^{3}}{\left|\phi \left(x;\omega \right)\right|}^{2}\;dx$$

is square of the $L_{2}$-norm of the field (for the concrete value of the random parameter ω). Thus, normalization (determining “descriptive → observational” correspondence) is with respect to field’s energy.

Physical variables of PCSFT are quadratic forms of fields. Each quadratic form on $H=L_{2}$ is determined by a Hermitian operator, $\hat{A}:H\to H.$ Hence, PCSFT variables have the form,

$$f_{A}\left(\phi \right)\left(\omega \right)=\langle \phi \left(\omega \right)|A|\phi \left(\omega \right)\rangle ,$$

where $\phi \left(\omega \right)\equiv \phi \left(x;\omega \right)\in L_{2}$ for each ω. Quadratic forms are elements of the descriptive theory (PCSFT) and Hermitian operators are elements of the observational theory.

Averages calculated in PCSFT coincide with averages calculated in QM. However, the range of values of a quadratic form does not coincide with the range of values of the corresponding quantum observable, Hermitian operator (cf. with descriptive theories of the Bell type).
